# Supplementary figures and images for: The effects of trans-chalcone and chalcone 4 hydrate on the growth of Babesia and Theileria
Source: PLoS Negl Trop Dis. 2019 May 24;13(5):e0007030. doi: 10.1371/journal.pntd.0007030 (PMC6534319; doi:10.1371/journal.pntd.0007030)

Growth of *B. bovis*

A

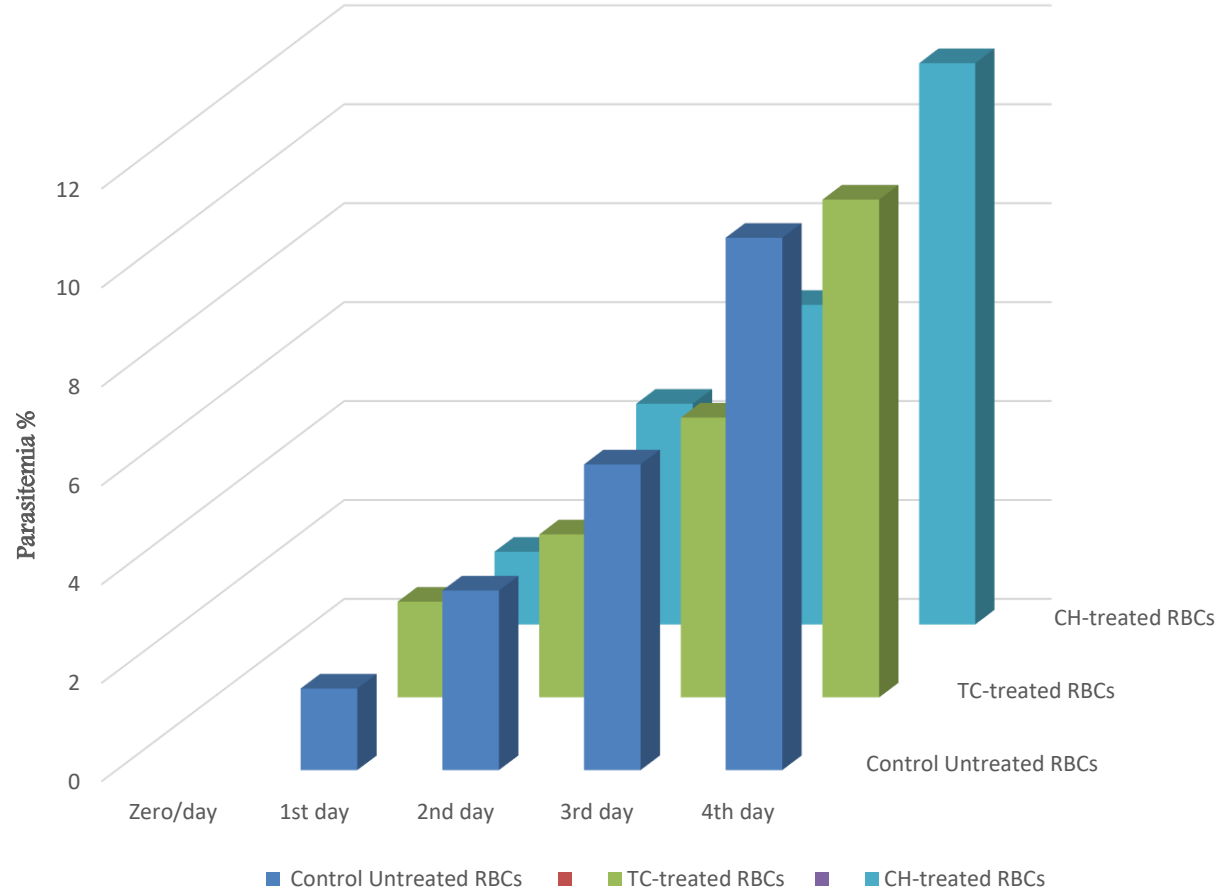

Growth of *T. equi*

B

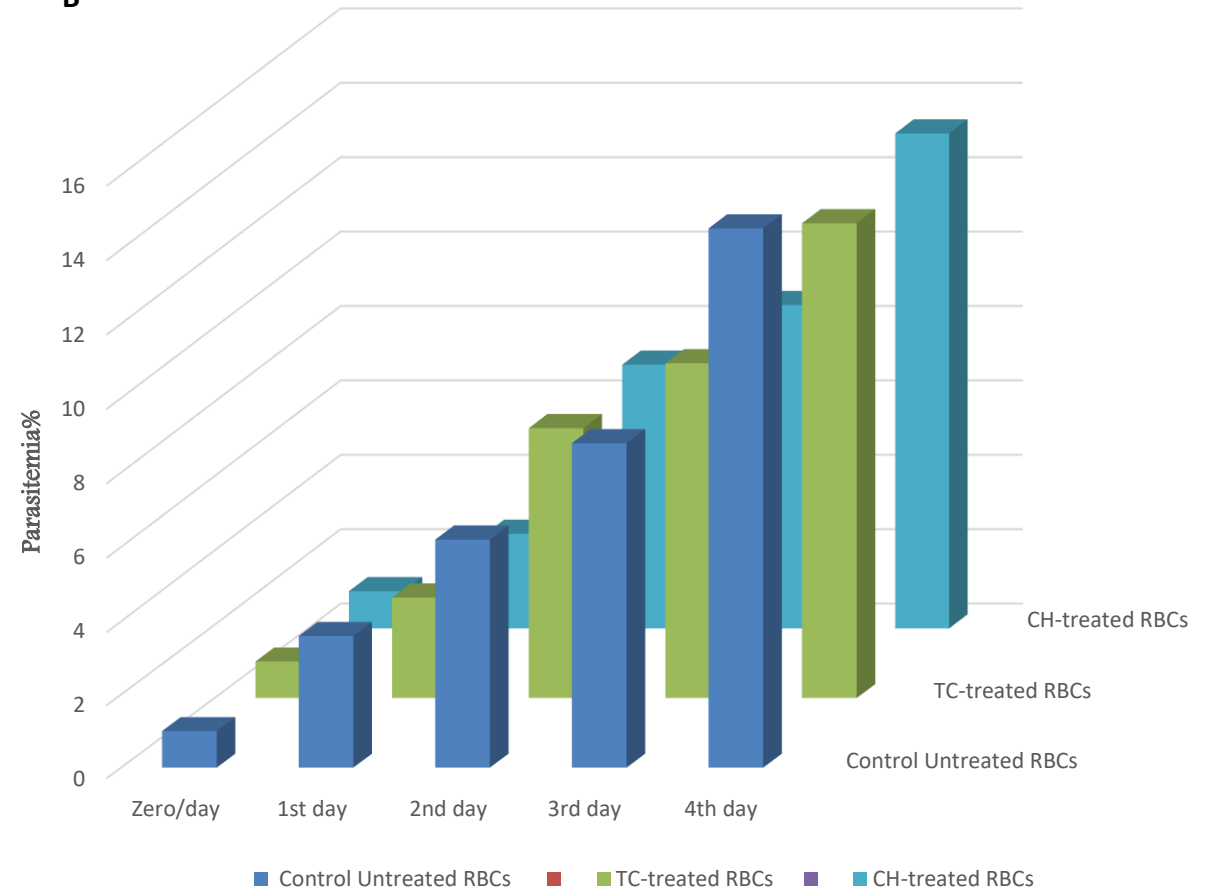

Supplement: S1 Fig — Growth of B. bovis with TC- or CH-treated bovine RBCs (A) and growth of T. equi with TC- or CH-treated horse RBCs (B). (PDF) [file pntd.0007030.s001.pdf]
