# Supplementary material for: The effects of trans-chalcone and chalcone 4 hydrate on the growth of Babesia and Theileria
Source: PLoS Negl Trop Dis. 2019 May 24;13(5):e0007030. doi: 10.1371/journal.pntd.0007030 (PMC6534319; doi:10.1371/journal.pntd.0007030)
Supplement: S2 Table — (DOCX) [file pntd.0007030.s003.docx]

**S2Table. Concentrations of *trans*-chalcone and chalcone hydrate combined with diminazene aceturate, atovaquone and clofazimine against *Babesia* and *Theileria* parasites *in vitro***

| **Parasite** | **Concentration**  (**µM**) | **Trans-chalcone** | **Chalcone hydrate** | **Diminazine aceturate** | **Atovaquone** | **Clofazimine** |
| --- | --- | --- | --- | --- | --- | --- |
| ***B. bovis*** | **C_1_** | 17.4 | 34.6 | 0.0875 | 0.00975 | 2.06 |
|  | **C_2_** | 34.8 | 69.2 | 0.175 | 0.0195 | 4.12 |
|  | **C_3_** | 69.6 | 138.4 | 0.35 | 0.039 | 8.24 |
|  | **C_4_** | 139.2 | 276.8 | 0.7 | 0.078 | 16.48 |
|  | **C_5_** | 278.4 | 553.6 | 1.4 | 0.156 | 32.96 |
|  |  |  |  |  |  |  |
| ***B. bigemina*** | **C_1_** | 8.33 | 15.225 | 0.17 | 0.17525 | 1.4325 |
|  | **C_2_** | 16.7 | 30.45 | 0.34 | 0.3505 | 2.865 |
|  | **C_3_** | 33.3 | 60.9 | 0.68 | 0.701 | 5.73 |
|  | **C_4_** | 66.6 | 121.8 | 1.36 | 1.402 | 11.46 |
|  | **C_5_** | 133.2 | 243.6 | 2.72 | 2.804 | 22.92 |
|  |  |  |  |  |  |  |
| ***B. divergens*** | **C_1_** | 16.2 | 20.575 | 0.1075 | 0.0095 | 3.4625 |
|  | **C_2_** | 32.4 | 41.15 | 0.215 | 0.019 | 6.925 |
|  | **C_3_** | 64.8 | 82.3 | 0.43 | 0.038 | 13.85 |
|  | **C_4_** | 129.6 | 164.6 | 0.86 | 0.076 | 27.7 |
|  | **C_5_** | 259.2 | 329.2 | 1.72 | 0.152 | 55.4 |
|  |  |  |  |  |  |  |
| ***B. caballi*** | **C_1_** | 4.725 | 6.975 | 0.0055 | 0.0255 | 1.9875 |
|  | **C_2_** | 9.45 | 13.95 | 0.011 | 0.051 | 3.975 |
|  | **C_3_** | 18.9 | 27.9 | 0.022 | 0.102 | 7.95 |
|  | **C_4_** | 37.8 | 55.8 | 0.044 | 0.204 | 15.9 |
|  | **C_5_** | 75.6 | 111.6 | 0.088 | 0.408 | 31.8 |
|  |  |  |  |  |  |  |
| ***T. equi*** | **C_1_** | 3.575 | 4.8 | 0.775 | 0.02375 | 0.72 |
|  | **C_2_** | 7.15 | 9.6 | 0.355 | 0.0475 | 1.44 |
|  | **C_3_** | 14.3 | 19.2 | 0.71 | 0.095 | 2.88 |
|  | **C_4_** | 28.6 | 38.4 | 1.42 | 0.19 | 5.76 |
|  | **C_5_** | 57.2 | 76.8 | 2.84 | 0.38 | 11.52 |

Note: ^a^ C_1_–C_5_ refers to the concentrations (**µM**) 0.25×IC_50_, 0.5×IC_50_, 1×IC_50_, 2 ×IC_50_, 4 ×IC_50_ of trans-chalcone, chalcone hydrate combined with diminazene aceturate, atovaquone, and clofazimine. Combined concentrations were based on the calculated IC_50_ values obtained from the *in vitro* fluorescence-based assay.
