# Supplementary material for: The effects of trans-chalcone and chalcone 4 hydrate on the growth of Babesia and Theileria
Source: PLoS Negl Trop Dis. 2019 May 24;13(5):e0007030. doi: 10.1371/journal.pntd.0007030 (PMC6534319; doi:10.1371/journal.pntd.0007030)
Supplement: S3 Table — (DOCX) [file pntd.0007030.s004.docx]

**S3 Table. Calculation of weighted average of combination Index values**

| **Weighted average CI values ^b^** | **Combination index values at** | | | | **Drug combinations ^a^** | **Parasites** |
| --- | --- | --- | --- | --- | --- | --- |
|  | **IC_95_** | **IC_90_** | **IC_75_** | **IC_50_** |  |  |
| 1.05573  1.06140 | 0.907  1.002 | 1.403  0.882 | 0.963  0.625 | 0.7943  2.710 | TC + DA  CH + DA | ***B. bovis*** |
| 1.0912  0.38743 | 1.503  0.374 | 0.768  0.411 | 0.991  0.357 | 0.614  0.4313 | TC + AQ  CH + AQ |  |
| 1.03190  1.04538 | 1.510  1.477 | 1.008  0.815 | 0.591  0.897 | 0.2078  0.3068 | TC + CF  CH + CF |  |
| 1.0588  1.0733 | 1.101  1.011 | 0.954  1.005 | 1.090  1.047 | 1.142  1.580 | TC +DA  CH +DA | ***B. bigemina*** |
| 0.07825  1.09269 | 0.022  1.050 | 0.025  1.209 | 0.073  0.777 | 0.4735  1.5459 | TC +AQ  CH + AQ |  |
| 0.11538  0.16805 | 0.103  0.222 | 0.118  0.010 | 0.011  0.293 | 0.3658  0.1765 | TC +CF  CH + CF |  |
| 0.51622  0.51622 | 0.501  0.433 | 0.418  0.319 | 0.359  0.572 | 1.1862  1.3292 | TC +DA  CH +DA | ***B. divergens*** |
| 0.97607  0.76130 | 0.977  0.853 | 0.989  0.701 | 0.786  0.981 | 1.3137  0.1360 | TC +AQ  CH + AQ |  |
| 0.72582  1.00823 | 0.703  1.073 | 0.777  1.303 | 0.541  0.810 | 1.0332  0.2613 | TC +CF  CH + CF |  |
| 0.14276  0.20318 | 0.008  0.172 | 0.165  0.372 | 0.118  0.052 | 0.6646  0.1238 | TC +DA  CH +DA | ***B. caballi*** |
| 0.22977  0.07987 | 0.119  0.053 | 0.111  0.091 | 0.282  0.103 | 0.9247  0.1077 | TC +AQ  CH + AQ |  |
| 0.73026  0.32754 | 0.601  0.473 | 0.608  0.113 | 0.697  0.192 | 1.6806  0.6604 | TC +CF  CH + CF |  |
| 0.11061  0.04491 | 0.122  0.010 | 0.111  0.001 | 0.132  0.020 | 0.0211  0.3661 | TC +DA  CH +DA | ***T. equi*** |
| 0.52253  0.10921 | 0.452  0.158 | 0.431  0.013 | 0.537  0.191 | 1.0503  0.0391 | TC +AQ  CH + AQ |  |
| 0.84954  0.48165 | 0.803  0.413 | 0.767  0.413 | 0.692  0.491 | 1.5984  0.9435 | TC +CF  CH + CF |  |

CI value, combination index value; IC_50_, 50% inhibition concentration; DA, diminazene aceturate; AQ, atovaquone. ^a^ Two-drug combination between *trans*-chalcone, chalcone hydrate with diminazene aceturate, and atovaquone at a concentration of approximately 0.25 x IC_50_, 0.5 x IC_50_, IC_50_, 2 x IC_50_, and 4 x IC_50_ (constant ratio). ^b^ The weighted average CI value was calculated with the formula [(1 x IC_50_) + (2 x IC_75_) + (3 x IC_90_) + (4 x IC_95_)]/10.
